# Cohen’s h for detection of disease association with rare genetic variants

**DOI:** 10.1186/1471-2164-15-875

**Published:** 2014-10-08

**Authors:** Shu-Hui Wen, Jih-I Yeh

**Affiliations:** Department of Public Health, College of Medicine, Tzu-Chi University, 701, Sec 3, Chung-Yang Rd, Hualien, 97004 Taiwan; Department of Molecular Biology and Human Genetics, Tzu-Chi University, 701, Sec 3, Chung-Yang Rd, Hualien, 97004 Taiwan; Department of Family Medicine, Buddhist Tzu-Chi General Hospital, 707, Sec 3, Chung-Yang Rd, Hualien, 97002 Taiwan

**Keywords:** Effect size, Cohen’s h, Odds ratio, Power, Rare variant

## Abstract

**Background:**

The power of the genome wide association studies starts to go down when the minor allele frequency (MAF) is below 0.05. Here, we proposed the use of Cohen’s h in detecting disease associated rare variants. The variance stabilizing effect based on the arcsine square root transformation of MAFs to generate Cohen’s h contributed to the statistical power for rare variants analysis. We re-analyzed published datasets, one microarray and one sequencing based, and used simulation to compare the performance of Cohen’s h with the risk difference (RD) and odds ratio (OR).

**Results:**

The analysis showed that the type 1 error rate of Cohen’s h was as expected and Cohen’s h and RD were both less biased and had higher power than OR. The advantage of Cohen’s h was more obvious when MAF was less than 0.01.

**Conclusions:**

Cohen’s h can increase the power to find genetic association of rare variants and diseases, especially when MAF is less than 0.01.

**Electronic supplementary material:**

The online version of this article (doi:10.1186/1471-2164-15-875) contains supplementary material, which is available to authorized users.

## Background

The prevailing hypothesis in genome-wide association studies (GWASs) of genetic diseases is “common disease, common variant” [1, 2]. The development of microarray based genotyping greatly accelerated GWASs [3–5] and lead to the identification of hundreds of genetic variants causing susceptibility to complex diseases. Most identified common variants confer relatively small risk (odds ratio (OR) at 1.1-1.5) and explain only a modest proportion concerning the heritability of these diseases [6, 7]. In contrast, most of the identified rare variants have ORs above 2 [8, 9]. This leads to the question of how the missing heritability can be explained and the search for the possible contribution by rare variants [1, 10–12]. One technical obstacle to GWAS on rare variants is the calling algorithm of microarray based genotyping. The reliability of genotyping calls drops when the minor allele frequency (MAF) falls below 5%. Recent improvement in genotype calling algorithms of microarrays and growing availability of next generation sequencing technology make rare variant searching feasible [13].

Many strategies have been developed to analyze disease-associated rare variants, e.g. the collapsing method [14, 15], the weighted approach [16–18], and regression-based analysis [19, 20]. Generally, these strategies emphasize the significance of rare variants by either analyzing a cluster on aggregate or setting larger weights on them. These studies focus on hypothesis testing with the aim of increasing the power of detecting associated rare variants. The variability of odds ratio increases at the lower end of MAF, e.g. MAF =0.001 [21–23]. Recent studies raised issues of the OR based analysis of GWAS [12, 21, 24, 25]. They predicted additional loci to be uncovered by more powerful GWAS for these studied traits and combined with published loci could explain around 15-20% of heritability of these traits.

The aim of this study was to examine the distributions and properties of Cohen’s h [26], and compared its performance in analyzing GWAS data with OR and RD using publicly available GWAS datasets as well as simulated datasets. We used the coronary artery disease (CAD) GWAS dataset from the Wellcome Trust Case Control Consortium (WTCCC) [4], as well as a sequencing-based T1D dataset. We determined the Cohen’s h equivalents to the OR for declaring a mild, moderate and large effect. To take advantage of the fact that the power of Cohen’s h does not depend on MAFs, we describe how to use Cohen’s h to evaluate the power and sample sizes required in rare variant studies. Other possible applications of Cohen’s h for such studies of rare variants are also discussed.

## Methods

### Theoretical properties of effect size measures: risk difference, Cohen’s h and odds ratio

For biallelic SNPs with minor allele A, and major allele a, the case (group D) and control (group ) populations had *n*_1_ and *n*_2_ allele counts, respectively. Let *p*_1_ = *P*(*A*|*D*) be the MAF of the case group and  be the MAF of the control group. These three ES measures are functions of MAFs from case and control groups. RD is defined as d = *p*_1_ - *p*_2_, and the estimator is , where  and  are the maximum likelihood estimators of *p*_1_ and *p*_2_, respectively. The definition for Cohen’s h is , and the estimator is . The allelic OR can be defined as , and the corresponding estimator is .

The derivations of the asymptotic distributions of estimators and their applications to the calculation of the p-values were described in Additional file 1. When the normality approximation fails due to extremely low MAF, the p-values were validated by the Fisher’s exact test. The performance of these ES measures were evaluated by accuracy, precision (i.e., bias and mean square error (MSE)), and type I error rate. Furthermore, statistical power was computed by assuming a balanced case–control design with independent cases and controls. The power formula for testing *H*_0_ : *p*_1_ - *p*_2_ = 0 *vs. H*_1_ : *p*_1_ - *p*_2_ ≠ 0 with a total of *n* independent cases and controls (i.e. *n*_1_ = *n*_2_ = *n*) , where *Φ* denotes the cumulative normal distribution and *z*_1 - *α*/2_ was the 100(1-α/2)^-th^ percentile. The power for *H*_0_ : *OR* = 1 *vs. H*_1_ : *OR* = *c* could be determined provided that the MAF in the control group, *p*_2_, and *c* were known according to the above formula where the MAF in the case group could be calculated as *p*_1_ = (*OR* * *p*_2_)/(*OR* * *p*_2_ - *p*_2_ + 1). In the case of Cohen’s h, the association test can be set as *H*_0_ : *h* = 0 *vs. H*_1_ : *h* = *δ*, and the power formula is . The sample size n was varied from 1,000 to 10,000 and the MAF in the control group was varied from 0.001 to 0.05. The *c* values for OR of 1.8, 2, and 3, and *δ* values of 0.08, 0.1, and 0.13 for Cohen’s h were considered for *p*_2_ < 0.05. We used a nominal significance level of 5 × 10^-8^ to calculate the statistical power of a GWAS.

### Simulations and data analysis

#### GWAS data from the WTCCC, quality control and filtering

We obtained genotyping data of 1,988 CAD patients and 3,004 shared controls (1,504 from 1,958 Birth Cohort Controls (58C) and 1,500 from UK Blood Services sample (NBS)) from the WTCCC archive. The majority of subjects were of European descent. All individuals were genotyped using Affymetrix GeneChip 500 K arrays. Details of the study samples were described in the original report [4]. We calculated RD, Cohen’s h and log(OR) using this dataset. The individuals dropped in the WTCCC study because of evidence of non-European descent or genotyping problems were excluded in the current analysis. A total of 1,926 subjects with CAD and 2,938 common controls were included for further analysis. We further dropped the SNPs with bad genotype calling, as suggested in the original report. The exclusion criteria for SNPs were (1) MAF in shared controls is less than 0.002, at which there were less than 3 individuals for any genotype, (2) call rate <95%, and (3) Hardy–Weinberg Equilibrium exact test P value <5.7*10^-7^ in shared controls and (4) allele frequency difference test based on two samples proportion test P value <5.7 × 10^-7^ between 58C and NBS. A total of 413,059 SNPs consisting of 52,220 (12.64%) rare SNPs (MAF < 0.05) and 360,839 (87.36%) common SNPs (MAF≧0.05) passed this filter.

#### Using simulation to estimate type I error rate and power

To assess statistical properties of ES measures in terms of bias, MSE, and type I error rate, we performed simulations of a pseudo case–control study using the two shared controls. We randomly selected 1,480 subjects from pooled shared controls as pseudo-cases and kept the remaining 1,458 samples as pseudo-controls. For every replication, we calculated the ES estimate by each measure and tested the association for each SNP. The bias was calculated as the mean deviation of estimates from 0 per replication, and the MSE was the mean of the square of the bias. The fraction of times that the p-values of the association tests were less than 0.05 was the empirical type I error rate. These three indices for rare and common variants on each autosome were shown in Additional file 2 (bias, MSE) and Additional file 3 (type I error rate).

To better compare the performance of Cohen’s h with other methods including Combined Multivariate and Collapsing Method (CMC) [15], Weighted Sum Statistic (WSS) [16] and Variable Threshold (VT) [17], we used simulated rare variants datasets generated by the SimRare program ([27], http://code.google.com/p/simrare/). SimRare uses the forward-time simulation program to generate sequence data. Evolution parameters used were: (1) an additive multi-locus model with selection coefficient distribution by Kryukov [28], (2) the mutation rate was 1.8×10^-8^, and (3) the effective population sizes were 8,100, 8,100, 7,900, 900,000 with 500, 10, 370 generations, respectively. Fifty replications of fixed gene lengths including 250, 500, 1,000, 2,000, and 5,000 base pairs were simulated. The longer gene length produced a larger number of rare variants. The corresponding mean numbers of rare variants were 46.2, 96, 187.5, 377.7 and 944.3. For the setting of risk simulations, we assume a model with a disease prevalence rate of 1% and 2,000 cases and 2,000 controls. The power was assessed at OR = 0.5 for protective mutations and OR = 3 for detrimental mutations with an additive mode of inheritance over 1,000 replications. For CMC, WSS and VT, p-values were obtained empirically through 1,000 permutations for each replication (i.e., gene-specific). For single-marker testing methods such as RD, Cohen’s h and OR, the smallest p-value for testing the rare variant was recorded for each replication. We defined the unadjusted power for single marker testing methods as the proportion of replicates with minimum p-values < =0.05. Furthermore, we used Bonferroni correction and the Benjamin-Hochberg procedure [29] to adjust for multiple testing.

#### Applying Cohen’s h to microarray and sequencing-based datasets

We compared the three measures described above on one microarray typed (CAD) [4] and one sequence based (T1D) [30] dataset. For the CAD dataset, the association tests were used separately for the analysis of rare variants (0.05 > MAF ≧0.002) and common variants. For each SNP, the magnitude of ES was estimated by RD, OR, and Cohen’s h. Moreover, we adjusted the significance level by Bonferroni correction at a p-value threshold of 1.2 × 10^-7^(0.05/41,3059). The sequence based T1D dataset was retrieved from http://www.sciencemag.org/content/early/2009/03/05/science.1167728/rel-suppl/62c4d688b3668c3c/suppl/DC1. A total of 179 rare variants (defined as MAF < 3%) in 10 candidate genes were used in the current study [30].

## Results

### Performance of ES measures: bias, MSE, type I error and power

**Figure 1:**
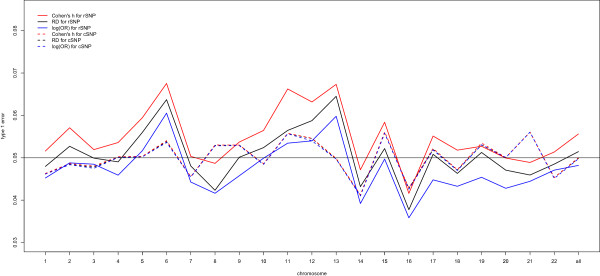
**Mean of empirical type I error rates for risk difference (RD), Cohen’s h and log(OR) in each autosome.**

Table 1 summarizes the accuracy (bias), precision (MSE) and type 1 error rate of RD, Cohen’s h, and OR for common and rare variants. Box-plots of the distributions of estimates of RD, Cohen’s h, and log(OR) for rare and common SNPs on each autosome are presented in Additional file 4 and Additional file 5. Among the 22 autosomes, mean biases and MSEs based on log(OR) of rare SNPs were larger than those of common SNPs, while RD and Cohen’s h obtained more similar estimates regardless of the MAFs. Thus, the OR would be more sensitive to changes in MAFs. Figure 1 presents the estimated type I error rates for RD, Cohen’s h, and log(OR) for common SNPs and rare SNPs. The performance of each measure of ES was very close for common SNPs in each autosome. The range of type I error rate for every ES measure was approximately (0.041, 0.056), and the average type I error rates for 22 autosomes were the same (0.05). As for rare SNPs, ranges of type I error rates of RD, Cohen’s h, and OR were (0.037, 0.065), (0.042, 0.068), and (0.036, 0.061), respectively. The value of the type I error rate for each ES measure was slightly larger than 0.05 at a few autosomes. The results indicated that every ES measure would probably produce slightly inflated type 1 error rates concerning the effect of rare variants in genetic association studies. Results from these simulations indicated that the estimate of OR for rare variant disease association might have greater bias and variability compared with RD and Cohen’s h. Equally important is the conclusion that the true significance may be missed by relatively large variation of OR estimates, followed by a loss of power to detect rare variants. This suggests potential utility of Cohen’s h for detecting rare variants associated with complex diseases.

**Table 1:** **Biases, MSEs and type I error rates for RD, Cohen’s h and OR**

ES	Type of SNP	No. SNPs	Bias	MSE	Min	Max	Type I error rate
RD	Common	360839	0.00004	0.00012	-0.046	0.044	0.050
	Rare	52220	0.00002	0.00001	-0.015	0.015	0.051
Cohen’s h	Common	360839	0.00008	0.00068	-0.103	0.097	0.050
	Rare	52220	0.00018	0.00072	-0.091	0.092	0.056
log(OR)	Common	360839	0.00021	0.00477	-0.346	0.346	0.050
	Rare	52220	0.00178	0.11395	-2.707	2.739	0.048

Table 2 showed the power of analyzing one marker at a time in comparison with methods (joint analysis methods) of jointly analyzing a group of mutations including CMC, WSS and VT in detecting disease associated genes. The power was lowest for single-marker tests and increased with the number of rare variants. When there were 96 variants within the gene on average, the unadjusted power was 0.465, 0.393, 0.521 and >0.878 for RD, OR, Cohen’s h and joint analysis methods, respectively. As the mean number of variants was increased from 96 to 377.7, the unadjusted power for the single-marker test increased to 0.88, 0.814, 0.918 and 1 for RD, OR, Cohen’s h and joint analysis methods, respectively. For these situations, the greatest power was observed in joint analysis methods, followed by Cohen’s h, which was always the largest of the three single-marker tests taking into account the adjustment for multiple testing. The results highlighted that for rare variants, Cohen’s h was a better association measure than RD and OR.

**Table 2:** **Empirical power for tests at nominal level 0.05 based on 1000 replicates**

Fixed gene length (bp)	Mean no of rare SNPs		RD	OR	Cohen’s h	CMC	WSS	VT
250	46.2					0.491	0.644	0.501
		Unadj.	0.142	0.107	0.178			
		BH	0.042	0.037	0.051			
		Bonf.	0.037	0.030	0.043			
500	96					0.878	0.931	0.882
		Unadj.	0.465	0.393	0.521			
		BH	0.106	0.080	0.135			
		Bonf.	0.087	0.064	0.113			
1000	187.5					0.992	0.998	0.992
		Unadj.	0.584	0.509	0.652			
		BH	0.136	0.109	0.162			
		Bonf.	0.121	0.083	0.141			
2000	377.7					1	1	1
		Unadj.	0.880	0.814	0.918			
		BH	0.254	0.194	0.306			
		Bonf.	0.211	0.143	0.256			
5000	944.3					1	1	1
		Unadj.	0.973	0.94	0.987			
		BH	0.370	0.265	0.451			
		Bonf.	0.305	0.191	0.388			

Unadj.: Without adjustment for multiple testing. BH: Benjamini-Hochberg procedure. Bonf.: Bonferroni correction.

### Statistical power required to detect disease association of rare SNPs based on Cohen’s h

**Figure 2:**
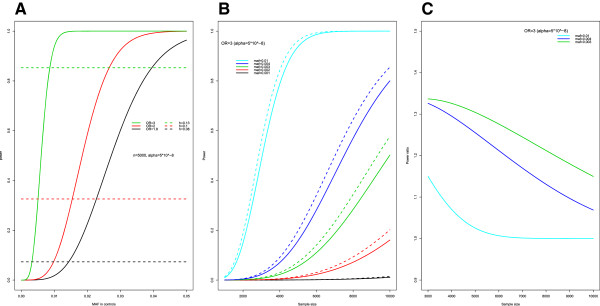
**Relationship between power and needed sample size based on OR and Cohen’s h for rare SNPs. (A)** Line plot shows the power estimated by OR (bold line) and Cohen’s h (dotted line) at the same threshold. **(B)** power curves given fixed OR =3 with corresponding Cohen’s h at varying MAF in controls. **(C)** Power ratio at varying MAF in controls and varying sample size.

Generally, the statistical power is related to the magnitude of the ES, the sample size, and the variance of the estimator of ES. The variance of each of the ES measures except Cohen’s h is related to the MAF in cases and controls (shown in Additional file 1). Accordingly, a MAF threshold was adopted to avoid limited power for SNPs with low MAF, and the threshold chosen also depended on the sample size of the study and the expected ES values. Figure 2A illustrated the relationship between the statistical power and the MAFs in the control group given similar magnitudes of ES for OR and Cohen’s h (n =5,000). Cohen’s h was more powerful than OR (Figure 2B). Even at a stringent significance level of *α* =10^-8^, the power of Cohen’s h remained higher than that of OR at *α* =5 × 10^-8^ for SNPs with MAF < 0.001 (data not shown).

For p_2_ ≤ 0.01, when the sample sizes increased from 1,000 to 10,000 and, we found that for all scenarios, the power of Cohen’s h remained higher than that of OR for the same ES measures (Figure 2B). For a SNP with OR =3 and p_2_ = 0.01, a total sample size of 4,060 (2,030 cases and 2,030 controls) was needed to achieve 80% statistical power to detect the effect at a genome-wide significance level of 5 × 10^-8^. However, the statistical power of Cohen’s h was approximately 85% with the same sample size. Additionally, the power ratio of the power based on Cohen’s h versus that of OR was consistently larger than 1 (Figure 2C). Hence, Cohen’s h was more powerful at identifying rare SNPs. The notable power gain of Cohen’s h at lower MAFs might contribute to the findings for rare SNPs.

### Analyses of rare SNPs in CAD data

**Figure 3:**
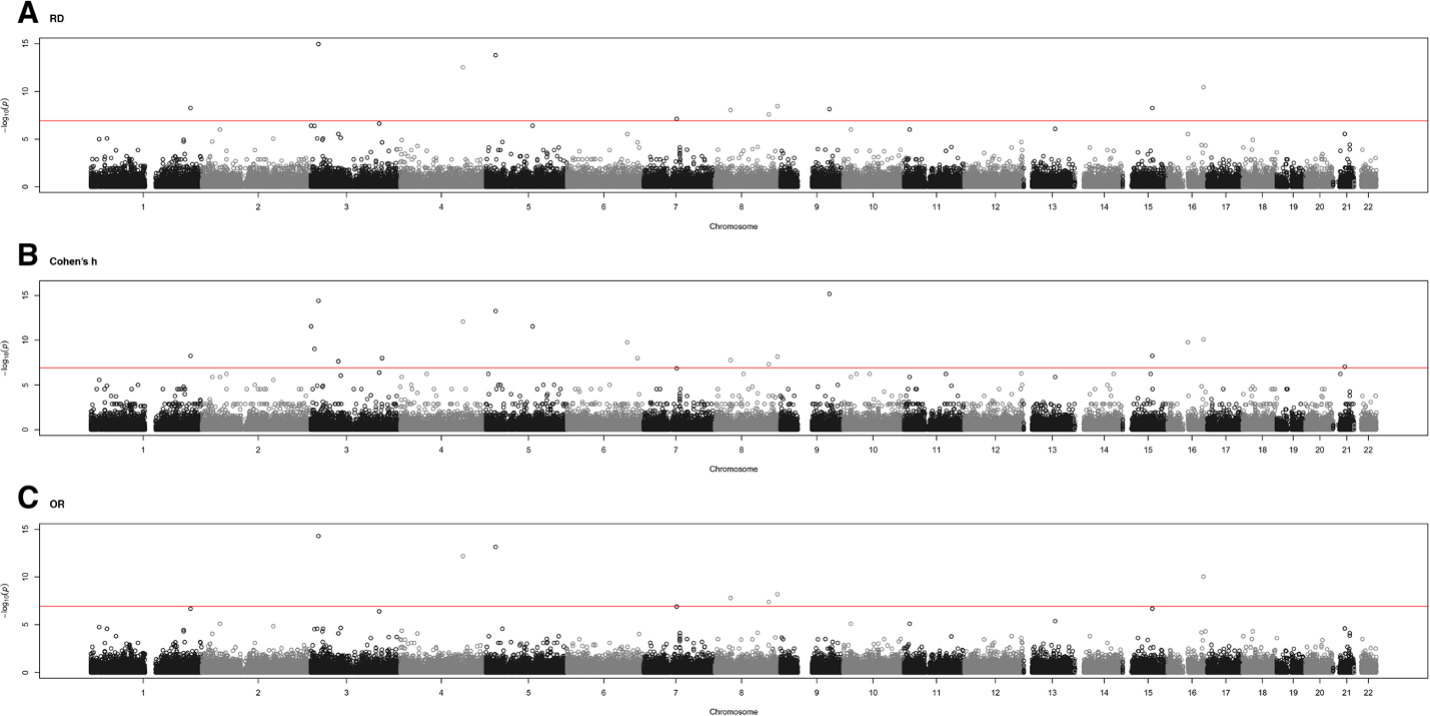
**Manhattan plot showing the significance of association between all rare SNPs and CAD.** For all panels, the genome-wide significance threshold of 0.05/403,089 is shown. Distributions of -log10 p-values for **(A)** risk difference, **(B)** Cohen’s h and **(C)** log(OR).

We performed single marker association tests using all three ES measures on the WTCCC CAD GWAS data. A total of 2,938 common controls and 1,926 cases with CAD were included in this study. We applied one single marker test at each of the 52,220 rare SNPs and 360,839 common SNPs separately. In addition, when the MAF in CAD patients was extremely low i.e. <0.002, the asymptotic assumption might not hold. In this case, the statistical significance of the p-values was validated by Fisher’s exact test. Bonferroni correction adjusted p-value of 0.05/413,059 was the criteria to declare genome-wide significance for any SNP (Figure 3). Table 3 summarized the number of significant SNPs, and genes that had been identified or validated for CAD based on OR, RD, and Cohen’s h, respectively. We found that among the 26 SNPs associated with CAD, 17 were on chromosome 9p21.3. The association of these regions with CAD was reported [4, 23]. All 3 ES measures identified the same 5 genes associated with CAD. The relevance of 4 of these genes, PLCL2, SAMD12-AS1, GAN, and MEF2NB-MEN2B, to intermediated cardiac phenotypes was reported [31–33].

**Table 3:** **Significant SNPs for CAD under different ES measures at genome-wide significance levels**

Type of SNPs	Significant SNPs	OR	RD	Cohen’s h
Common	Number	26	26	26
	Median	1.327	0.065	0.134
	Range	(0.757, 6.104)	(-0.063, 0.354)	(-0.129, 0.795)
Rare	Number	9	13	18
	Median	2.144	0.021	0.119
	Range	(1.88, 2.41)	(-0.009, 0.038)	(-0.167, 0.179)

Interestingly, when analyzing rare SNPs, the numbers of significant SNPs were different for each ES measure. Cohen’s h identified most SNPs, followed by RD, then OR. Compared to the OR results, there were 4 and 9 more SNPs detected by RD and Cohen’s h, respectively (Table 3). The substantial power gain of Cohen’s h leads to the identification of more significant rare SNPs. Some SNPs which were reported to be associated with CAD, such as rs17146094 (within EIF4H gene), and rs6674781 (near rs6671793), were identified by RD and Cohen’s h but not OR [5, 34].

Surprisingly, 6 further SNPs detected only by Cohen’s h, consistent with the findings based on Fisher’s exact test, were located in regions that are known to be associated with CAD, cholesterol, and arteries (Table 4). This finding again shows the ability of Cohen’s h to identify potential rare SNPs associated with disease and other intermediate disease phenotypes. Although SNPs implicated by our analysis are tightly correlated with other validated relevant SNPs in the region and are likely to be in linkage disequilibrium with the causal variant, most of the significant rare SNPs remain directly correlated to CAD. Caution should be taken on interpreting these results from the rare-variant analysis of the three WTCCC datasets as the reliability of current microarray based genotyping still needs improvement for MAF <5%.

**Table 4:** **Replication of rare SNPs showing statistically significant effect at genome-wide significance levels (1.2 × 10** ^**-7**^ **) for CAD**

Chr	ES	SNP	Nearest gene or SNP	Location	MAF in controls	MAF in cases	OR	P-value	RD	P-value	Cohen’s h	P-value	Exact test P-value ^b^	Association of SNP or proxy with other cardiovascular phenotypes
Genes	within	associated	interval											
3	All	rs17042882	PLCL2	3p24.3	0.028	0.061	2.255	4.88 × 10^-15^	0.033	1.11 × 10^-15^	0.163	4.00 × 10^-15^	---	Heart failure, Arthritis
3	h	rs16827563	VEPH1	3q24-q25	0.005	0	NA	NA	-0.005	2.18 × 10^-5^	-0.119	1.02 × 10^-8^	7.3 × 10^-7^	Carotid artery disease, Diabetes Mellitus
7	RD	rs17146094	EIF4H	7q11.23	0.017	0.034	2.036	1.27 × 10^-7^	0.017	7.15 × 10^-8^	0.109	1.32 × 10^-7^	---	CAD
8	All	rs16891338	SAMD12-AS1	8q24.12	0.023	0.043	1.908	4.11 × 10^-8^	0.02	2.50 × 10^-8^	0.113	4.66 × 10^-8^	---	Blood Pressure
8	All	rs16908145	FLJ45872	8q24.23	0.022	0.043	1.998	6.54 × 10^-9^	0.021	3.46 × 10^-9^	0.12	7.08 × 10^-9^	---	
15	RD, h	rs7163007	MAP2K5	15q23	0.002	0.011	5.551	2.13 × 10^-7^	0.009	5.33 × 10^-9^	0.121	5.85 × 10^-9^	---	BMI, Diabetes Mellitus
16	All	rs16955238	GAN	16q24.1	0.022	0.046	2.143	8.91 × 10^-11^	0.024	3.41 × 10^-11^	0.135	8.53 × 10^-11^	---	Cholesterol
16	h	rs7197337	ANKRD26P1	16q11.2	0.006	0	NA	NA	-0.006	2.88 × 10^-6^	-0.132	1.76 × 10^-10^	2.3 × 10^-8^	
19	All	rs11671119	MEF2B MEF2NB	19p13.11	0.033	0.071	2.239	0	0.038	0	0.174	0	---	Diabetes Mellitus
SNPs near associated SNPs within 500 kb														
1	RD, h	rs6674781	rs6671793	2^a^	0.002	0.011	5.55	2.13 × 10^-7^	0.009	5.33 × 10^-9^	0.121	5.85 × 10^-9^	---	Coronary disease
3	h	rs17064749	rs7615788	10^a^	0.008	0.001	0.124	8.36 × 10^-5^	-0.007	2.84 × 10^-6^	-0.116	2.28 × 10^-8^	4.2 × 10^-7^	Cholesterol
3	h	rs10510375	rs1450097	400^a^	0.009	0.001	0.11	2.97 × 10^-5^	-0.008	4.03 × 10^-7^	-0.127	9.67 × 10^-10^	2.2 × 10^-8^	Cholesterol, HDL
3	h	rs6805861	rs10510197	250^a^	0.007	0	NA	NA	-0.007	3.84 × 10^-7^	-0.145	2.92 × 10^-12^	1.2 × 10^-9^	Cholesterol, HDL
4	All	rs890447	rs97669522	25^a^	0.043	0.078	1.883	6.49 × 10^-13^	0.035	3.09 × 10^-13^	0.148	8.35 × 10^-13^	---	CAD
5	All	rs159171	rs10520872	500^a^	0.025	0.055	2.27	6.88 × 10^-14^	0.03	1.62 × 10^-14^	0.156	5.51 × 10^-14^	---	Cholesterol, LDL
5	h	rs41349146	rs2431337	500^a^	0.007	0	NA	NA	-0.007	3.84 × 10^-7^	-0.145	2.92 × 10^-12^	1.2 × 10^-9^	Arteries
6	h	rs41518850	rs12190287	300^a^	0.006	0	NA	NA	-0.006	2.88 × 10^-6^	-0.132	1.76 × 10^-10^	2.3 × 10^-8^	CAD
6	h	rs4398751	rs9397922	150^a^	0.005	0	NA	NA	-0.005	2.18 × 10^-5^	-0.119	1.02 × 10^-8^	7.3 × 10^-7^	Lipoprotein
8	All	rs16883114	rs10503973	200^a^	0.021	0.041	1.993	1.57 × 10^-8^	0.02	8.57 × 10^-9^	0.117	1.69 × 10^-8^	---	Cholesterol, LDL
9	RD, h	rs12343115	rs2149998	300^a^	0.009	0	NA	NA	-0.009	6.97 × 10^-9^	-0.167	6.66 × 10^-16^	3.6 × 10^-12^	Myocardial Infarction
18	All	rs41477147	rs10502528	150^a^	0.028	0.065	2.413	0	0.037	0	0.179	0	---	Arteries
		(rs1595963)												
21	h	rs7276641	rs2829644	300^a^	0.01	0.002	0.198	2.50 × 10^-5^	-0.008	2.81 × 10^-6^	-0.111	8.91 × 10^-8^	---	Coronary disease

^a^denotes the physical distance (in kb) to the nearest validated SNP. ^b^Fisher’s exact test is only required when the asymptotic assumption does not hold. NA: not available; Chr., chromosome; MAF, minor allele frequency; location according to NCBI Build 37.5; Association of SNP or proxy with other cardiovascular phenotypes was based on the HuGE Navigator database (http://hugenavigator.net/HuGENavigator/startPagePubLit.do), dbSNP (NCBI website: http://www.ncbi.nlm.nih.gov/projects/SNP/) and MalaCards (http://www.malacards.org/pages/whatsmalacards).

### Application to sequencing based T1D data

We also tested Cohen’s h on sequencing data. There were 179 rare variants in 10 candidate genes previously studied in connection with T1D. Four SNPs (rs35667974, rs35337543, ss107794687, and ss107794688) which were shown to be associated with T1D in the previous study were also identified by RD, Cohen’s h and OR. Another SNP, ss107794716 residing within the AIRE gene, was identified by both of RD and Cohen’s h but not OR (RD = 0.006, p = 0.034; Cohen’s h = 0.106, p = 0.019; OR = 7.04, p = 0.068). The association of this region and the AIRE gene with T1D was documented [35–37]. In addition, the magnitudes of p-values of significant rare variants obtained from Cohen’s h are the smallest. The results indicates that Cohen’s h are more likely to identify associated rare variants compared to OR.

### Distribution of ES values for rare variants based on CAD data

**Figure 4:**
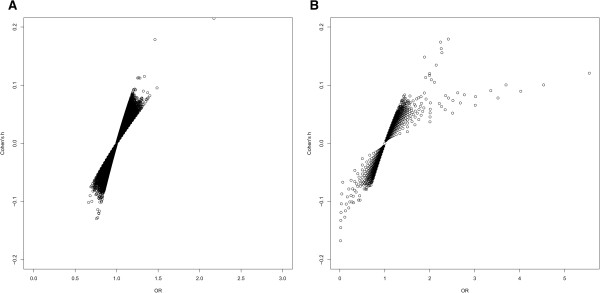
**Scatter plot of OR and Cohen’s h for rare and common SNPs in CAD. (A)** common SNPs and **(B)** rare SNPs. Despite the seemingly more outliers using the OR criteria, more outliers using Cohen’s h criteria were statistically significant.

The distributions of ES values for rare variants reflected the magnitude of rare variant-disease associations. We further explored the ES distributions of Cohen’s h and OR with regard to robustness and interpretation using the GWAS data. The two panels in Figure 4 showed the scatter plot of OR and Cohen’s h for the rare and common SNPs, respectively, in the CAD dataset. The ranges of ORs among rare SNPs were obviously broader than those of common SNPs. For Cohen’s h, the ranges were comparable for rare and common SNPs. This indicated that Cohen’s h is more robust at lower MAF compared to OR.

Thresholds of Cohen’s h for the evaluation of small, medium, and large differences between proportions were previously recommended as 0.2, 0.5, and 0.8, respectively [26]. However, the proportion of values of Cohen’s h larger than 0.2 was extremely low from our analysis of GWAS data (Figure 4). Therefore, we determined the empirical thresholds of Cohen’s h comparable to commonly used cut points for OR in terms of mild, moderate and large effects. The relationship between OR and Cohen’s h can be derived as  given the MAF of the control group, *p*_2_, and the OR. We found that the relationship between the MAF and Cohen’s h for rare SNPs was similar to that for common SNPs. As MAF increases, the magnitudes of Cohen’s h turned out to be greater at fixed OR. As such, we determined thresholds for Cohen’s h according to the average value of varying MAFs. For common SNPs, the values at 1.2 and 1.5 for OR are comparable to thresholds at 0.075 and 0.15 for Cohen’s h, respectively. As for rare SNPs, Cohen’s h had thresholds 0.05 and 0.1 corresponding to mild and moderate effects of OR at 1.5 and 2. One can choose dynamic thresholds for Cohen’s h with varying MAFs; however, this approach is not practical in comparison to using the p value of the calculated h. Using the above criteria, we further subdivided all common SNPs into the mild, moderate, and larger effect categories (Table 5). The proportions of each category estimated from the OR and Cohen’s h values for common SNPs resulted in good agreement (see Additional file 6). For common SNPs, the proportion of large ES (i.e., |log(OR)| > log(1.5)) falls in the range of (0.001%, 0.033%) for CAD, CD (data not shown), and RA (data not shown). This finding is consistent with that obtained from Cohen’s h (i.e., |h| >0.15). Notably, there was a trend that SNPs with lower MAF were more likely to have moderate to large ESs by either measure. However, for rare SNPs, the proportions of the three categories did not align (see Additional file 7). The proportion of large effects based on OR (i.e. |log(OR)| > log(2)) rose to 6.296% in CAD, revealing that approximately 3,288 rare SNPs have large ESs. In contrast, the estimates from Cohen’s h with comparably large ESs to OR, lead to markedly fewer, 42 in total, rare SNPs.

**Table 5:** **Proportions of SNPs with mild, moderate, and large effect for CAD GWAS data**

Type of	No. of	Mild effect (%)		Moderate effect (%)		Large effect (%)	
SNPs	SNPs	OR	Cohen’s h	OR	Cohen’s h	OR	Cohen’s h
Common	360839	99.453	99.936	0.546	0.063	0.001	0.001
Rare	52220	69.772	73.416	23.932	26.505	6.296	0.079

The respective thresholds of ORs for mild, moderate and large effect at common SNPs were |log(OR)|≦log(1.2), log(1.2) < |log(OR)|≦log(1.5), and |log(OR)| > log(1.5), whereas Cohen’s h had respective thresholds of |h|≦0.075, 0.075 < |h|≦0.15, and |h| > 0.15. The respective thresholds of ORs for mild, moderate and large effect at rare SNPs were |log(OR)|≦log(1.5), log(1.5) < |log(OR)|≦log(2), and |log(OR)| > log(2), whereas Cohen’s h had respective thresholds of |h|≦0.05, 0.05 < |h|≦0.1, and |h| > 0.1.

## Discussion

Despite the hundreds of common genetic variants associated with complex diseases identified by GWAS, only a small fraction of heritability of most common complex genetic diseases are explained by these genes. Currently, an increasing number of studies are focusing on rare disease-associated variants that might shed light on the issue of missing heritability. The power of GWAS falls steeply with MAFs for values <0.01. We compared Cohen’s h with OR on simulated and real data. Our results supported the following conclusions. First, estimates of the ES measures were biased at low MAF regardless of the method used. The estimates of log(OR) were more biased and exhibited greater MSE for rare variants than RD and Cohen’s h, as was reported [21, 24]. In contrast to previous simulation studies [21, 22, 24], we found slightly increased false positive associations for rare variants (Figure 1) exceeding the nominal level (5%). Possible strategies to address this problem included the adoption of a more stringent significance level to prevent inflated false positive results and to aggregate multiple rare SNPs to avoid the burden of multiple testing [38]. We noticed that single marker testing was not as efficient as methods that jointly analyze a group of mutations such as CMC, WSS and VT. Some studies had demonstrated that CMC, WSS and VT would encounter the loss of power when the direction of effects in the combined variants is not consistent, or when a small fraction of variants are associated with disease, as compared to single marker testing [39, 40]. Hence, we recommend using Cohen’s h for screening purpose to uncover SNPs that might be overlooked by the OR or RD based statistic commonly used in GWAS. Once the candidate genes were flagged, more sophisticated statistical methods and re-sequencing of these potential target regions and more would be needed for validation.

Second, we compared the empirical distributions of ORs and Cohen’s h for common and rare variants corresponding to the null hypothesis of WTCCC GWAS with ~2000 cases and 3000 controls. To the best of our knowledge, most studies used significant or susceptible SNPs from GWAS findings to examine the distributions of ORs for common and rare variants [8, 12, 25]. However, very weak genetic effects would likely be missed by studies using only significant SNPs. In our study, the empirical distributions of ORs could be useful for setting realistic conditions related to the OR for rare variants in future simulation studies because the vast majority of studies typically utilize the OR as the ES measure. A quick search in PubMed using “Cohen’s h” did not find any genetic association studies. To our knowledge, the first paper that mentioned the application of Cohen’s h for rare variant was Evangelou & Ioannidis [41]. Our study provided supporting evidence that the application of Cohen’s h for rare variant analysis was appealing. Additionally, we made an evaluative judgment on whether the estimated value of Cohen’s h should be considered mild, moderate, or large to improve its interpretation. As was widely known, low frequency SNPs had moderate-to-large effects (compared to common SNPs,) based on the OR and Cohen’s h. However, for SNPs with MAFs between 0.002 and 0.05, the percentages of large effects based on OR (6.296%) were much greater than those obtained using Cohen’s h (0.079%). We argued that the relative greater bias of OR based estimates might be responsible for this large difference. The most common argument against the use of data transformation is the problem of interpretability in effect size estimation. The impression of imperfect correlation between Cohen’s h and effect size needs further study.

Third, our finding also showed that Cohen’s h could uncover rare disease-associated variants missed by OR based analysis. The arcsine square root transformation stabilized Cohen’s h so its asymptotic variance did not depend on the allele frequency. Accordingly, its power to detect a genetic association was relatively robust at low MAF. The data presented here suggest that test based on Cohen’s h is an appropriate substitute for OR (Table 4). The vast majority of the rare disease-associated variants identified by OR was detrimental. On the contrary, one was more likely to discover both risk and protective variants using Cohen’s h. Caution should be taken on interpreting these results from the rare-variant analysis of the three WTCCC datasets as the reliability of current microarray based genotyping still needed improvement for MAF <5%. Next-generation sequencing technologies, such as the 1000 Genome project, will identify many more variants with very low MAFs; thus, the application of Cohen’s h is appropriate. Additionally, estimating and presenting Cohen’s h facilitates future meta-analysis of GWAS data [41]. However, further studies are needed to address the slight bias and to control the false positive rates associated with the analysis of rare variants. In this situation, all other methods suffer from inflated type I error rates. Thus, alternative methods will need to be developed based on Cohen’s h to account for the increased numbers of false positives. There are likely several factors that contribute to the inflated type I error rates, such as population stratification and linkage disequilibrium among rare variants. One possible direction is to use methods to jointly analyze a group of mutations within a gene, or functional unit, as performed in previous studies [14–20]. Additional studies are required to delineate the optimal application and interpretation of results based on Cohen’s h.

## Conclusions

Using simulated and publically available data, our results suggested that Cohen’s h, a difference-type measure based on the arcsine square root transformation of minor allele frequencies, was less biased and substantially more powerful than OR in detecting the association of rare variants and complex genetic diseases. Our method offers a useful option for researchers who wish to quantify rare variants associated with diseases.

## Electronic supplementary material

Additional file 1: **Additional information of sampling distributions of RD, Cohen’s h and OR.** (PDF 246 KB)

Additional file 2: **Biases and MSEs for RD, Cohen’s h and log(OR) for 22 chromosomes.** (PDF 116 KB)

Additional file 3: **Type I error rates for RD, Cohen’s h and log(OR) for 22 chromosomes.** (PDF 91 KB)

Additional file 4: **Box-plot of effect sizes for rare SNPs based on two shared controls in WTCCC data.** Panel A: RD; Panel B: Cohen’s h; Panel C: log(OR). (PDF 262 KB)

Additional file 5: **Box-plot of effect sizes for common SNPs based on two shared controls in WTCCC data.** Panel A: RD; Panel B: Cohen’s h; Panel C: log(OR). (PDF 353 KB)

Additional file 6: **Proportions of mild, moderate, and large effect for common SNPs in CAD.** Grey bar represents OR and Black bar denotes Cohen’s h. (PDF 254 KB)

Additional file 7: **Proportions of mild, moderate, and large effect for rare SNPs in CAD.** Grey bar represents OR and Black bar denotes Cohen’s h. (PDF 250 KB)

## Abbreviations

MAF: Minor allele frequency
SNP: Single nucleotide polymorphism
RD: Risk difference
OR: Odds ratio
GWAS: Genome-wide association study
ES: Effect size
CAD: Coronary artery disease
WTCCC: Wellcome Trust Case Control Consortium
58C: 1958 Birth Cohort Controls
NBS: UK Blood Services sample
MSE: Mean square error
CMC: Combined Multivariate and Collapsing Method
WSS: Weighted sum statistic
VT: Variable threshold.
