# Supplementary material for: Cohen’s h for detection of disease association with rare genetic variants
Source: BMC Genomics. 2014 Oct 8;15(1):875. doi: 10.1186/1471-2164-15-875 (PMC4198687; doi:10.1186/1471-2164-15-875)
Supplement: Supplementary file 1 — Additional file 1: Additional information of sampling distributions of RD, Cohen’s h and OR. (PDF 246 KB) [file 12864_2014_6546_MOESM1_ESM.pdf]

Additional file 1 Additional information of sampling distributions of RD, Cohen's h and OR.

The asymptotic distribution for each of RD, Cohen's h and log(OR) can be derived by using the multivariate delta method. Suppose a random vector $\mathrm{X} = (\mathrm{X}_1, .., \mathrm{X}_\mathrm{p})$ has a mean vector $\mu = (\mu_1, ..., \mu_p)$ and a variance-covariance matrix Σ. Then, for a transformation of X is $g(X)$, the asymptotic distribution will be asymptotic normal $N(\mu_g, \sigma_g^2)$ where $\mu_g = g(\mu)$ and $\sigma_g^2 = D\Sigma D^t$, where D is the matrix of partial derivatives of $g(X)$ with respect to X and D^t denotes the transpose matrix of D. These three ES measures can be considered as a function of MAFs from case and control group. The joint distribution of estimates of MAFs at case and control group is

$$\begin{pmatrix} \hat{p}_1 \\ \hat{p}_2 \end{pmatrix} \sim N\left(\begin{pmatrix} p_1 \\ p_2 \end{pmatrix}, \begin{bmatrix} \frac{p_1(1-p_1)}{n_1} & 0 \\ 0 & \frac{p_2(1-p_2)}{n_2} \end{bmatrix} \right)$$

. Firstly, we derive the asymptotic distribution of the estimate for RD, denoted as $\hat{d} = \hat{p}_1 - \hat{p}_2$. Let $g(\hat{p}_1, \hat{p}_2) = \hat{p}_1 - \hat{p}_2$, the expectation value of $\hat{p}_1 - \hat{p}_2$ is $E(g(p_1, p_2)) = p_1 - p_2$ and variance is

$$Var(\hat{p}_1 - \hat{p}_2) = (1, -1) \times \begin{bmatrix} \frac{p_1(1-p_1)}{n_1} & 0 \\ 0 & \frac{p_2(1-p_2)}{n_2} \end{bmatrix} \times \begin{pmatrix} 1 \\ -1 \end{pmatrix} = \frac{p_1(1-p_1)}{n_1} + \frac{p_2(1-p_2)}{n_2}.$$

Hence $\hat{d}$ is asymptotically distributed as a normal distribution $N\left(d, \frac{p_1(1-p_1)}{n_1} + \frac{p_2(1-p_2)}{n_2}\right)$. The genetic association test based on RD can be defined as H_0:d =0 vs. H_1:d ≠0, and the corresponding test statistic is

$$\frac{\hat{d}}{\sqrt{\hat{p}(1-\hat{p})\times(\frac{1}{n_1}+\frac{1}{n_2})}}$$ where $\hat{p}=\frac{(n_1\hat{p}_1+n_2\hat{p}_2)}{n_1+n_2}$ is the MAF among whole ample.

Next, for Cohen's h, the estimator is $\hat{h}=g(\hat{p}_1,\hat{p}_2)=2\arcsin(\sqrt{\hat{p}_1})-2\arcsin(\sqrt{\hat{p}_2})$. According to the delta method, the expectation of Cohen's h is $E(g(p_1,p_2))=2\arcsin(\sqrt{p_1})-2\arcsin(\sqrt{p_2})$ and the variance is

$$Var(g(\hat{p}_1,\hat{p}_2))=(\frac{1}{\sqrt{p_1(1-p_1)}},-\frac{1}{\sqrt{p_1(1-p_1)}})\times\begin{bmatrix}\frac{p_1(1-p_1)}{n_1} & 0\\ 0 & \frac{p_2(1-p_2)}{n_2}\end{bmatrix}\times\begin{pmatrix}\frac{1}{\sqrt{p_1(1-p_1)}}\\ -\frac{1}{\sqrt{p_1(1-p_1)}}\end{pmatrix}$$

$=\frac{1}{n_1}+\frac{1}{n_2}$. The asymptotic distribution for $\hat{h}$ is $N\left(h,\frac{1}{n_1}+\frac{1}{n_2}\right)$. The test statistic based on the Cohen's h for $H_0{:}h=0$ vs. $H_1{:}h\neq 0$ is $\frac{\hat{h}}{\sqrt{\frac{1}{n_1}+\frac{1}{n_2}}}$. Finally, we derive the asymptotic distribution for log(OR) as below. The estimator for log(OR) is $\ln(\hat{OR})=g(\hat{p}_1,\hat{p}_2)=\ln(\frac{\hat{p}_1/(1-\hat{p}_1)}{\hat{p}_2/(1-\hat{p}_2)})$ and the expectation is $E(g(p_1,p_2))=\ln(\frac{p_1/(1-p_1)}{p_2/(1-p_2)})=\ln(OR)$. The variance is

$$Var(g(\hat{p}_1,\hat{p}_2))=(\frac{1}{p_1(1-p_1)},\frac{1}{p_2(1-p_2)})\times\begin{bmatrix}\frac{p_1(1-p_1)}{n_1} & 0\\ 0 & \frac{p_2(1-p_2)}{n_2}\end{bmatrix}\times\begin{pmatrix}\frac{1}{p_1(1-p_1)}\\ \frac{1}{p_2(1-p_2)}\end{pmatrix}$$

$=\frac{1}{n_1p_1(1-p_1)}+\frac{1}{n_2p_2(1-p_2)}$. Therefore, the asymptotic distribution of $\ln(\hat{OR})$ is $N\left(\ln(OR),\frac{1}{n_1p_1(1-p_1)}+\frac{1}{n_2p_2(1-p_2)}\right)$. The test statistic for genetic association

based on log(OR) is $\dfrac{\ln(O\hat{R})}{\sqrt{\dfrac{1}{n_1\hat{p}_1(1-\hat{p}_1)}+\dfrac{1}{n_2\hat{p}_2(1-\hat{p}_2)}}}$.
