# Supplementary material for: Cohen’s h for detection of disease association with rare genetic variants
Source: BMC Genomics. 2014 Oct 8;15(1):875. doi: 10.1186/1471-2164-15-875 (PMC4198687; doi:10.1186/1471-2164-15-875)
Supplement: Supplementary file 2 — Additional file 2: Biases and MSEs for RD, Cohen’s h and log(OR) for 22 chromosomes. (PDF 116 KB) [file 12864_2014_6546_MOESM2_ESM.pdf]

Additional file 2: Biases and MSEs for RD, Cohen's h and log(OR) for 22 chromosomes.

Chr.	no. of rSNP	Bias $\times 10^4$			MSE $\times 10^4$			no. of cSNP	Bias $\times 10^4$			MSE $\times 10^4$		
		RD	Cohen's h	log(OR)	RD	Cohen's h	log(OR)		RD	Cohen's h	log(OR)	RD	Cohen's h	log(OR)
1	4399	-0.3	1.2	74.1	0.1	7.2	1165.1	28753	0.5	1.8	5.7	1.2	6.6	46.4
2	4341	0.0	5.3	99.2	0.1	7.2	1130.9	30271	2.3	5.8	15.5	1.2	6.7	46.1
3	3484	-1.0	-3.3	-14.8	0.1	7.3	1155.7	25113	-2.4	-5.8	-15.2	1.2	6.6	44.7
4	3506	-0.3	-2.7	-55.3	0.2	7.4	1116.2	23447	1.7	5.8	20.5	1.2	6.7	47.2
5	3462	-0.3	0.4	29.5	0.1	7.3	1042.3	24020	3.4	8.3	21.3	1.2	6.8	46.9
6	3138	0.4	1.3	-26.4	0.2	7.3	966.3	24026	-0.7	-0.8	1.2	1.2	7.0	48.2
7	2641	1.3	8.4	60.8	0.1	7.0	1073.4	19504	0.4	1.3	4.8	1.2	6.6	45.7
8	2835	2.3	13.7	62.3	0.1	6.9	1091.7	20353	1.4	2.9	4.9	1.2	7.0	49.0
9	2514	1.3	7.9	37.8	0.1	7.3	1145.5	17134	0.5	2.3	10.3	1.2	7.1	49.6
10	3205	0.8	4.8	18.0	0.2	7.4	1137.4	20923	-4.0	-10.7	-29.3	1.2	6.8	49.1
11	2656	1.3	5.6	25.4	0.1	7.6	1600.0	19456	1.2	3.9	12.4	1.2	7.1	49.3
12	2722	-1.3	-7.8	-27.3	0.2	7.9	1147.2	18370	-1.2	-3.7	-11.6	1.2	7.0	49.2
13	2124	0.2	8.0	188.2	0.2	8.2	1073.8	14063	0.0	-0.3	-1.8	1.2	6.7	47.6
14	1759	0.1	-1.5	-13.9	0.1	6.8	1166.2	11421	-1.4	-2.3	-1.9	1.1	6.3	43.4
15	1610	-1.6	-1.0	166.7	0.1	7.3	1218.4	10287	1.3	2.9	6.5	1.2	7.0	48.2
16	1563	0.3	-1.0	-36.3	0.1	6.5	1053.6	11015	-5.4	-13.9	-38.1	1.1	6.4	44.0
17	1160	-5.3	-29.2	-135.9	0.2	7.6	1294.9	8257	5.8	14.7	38.8	1.2	7.0	47.1
18	1641	-1.1	-8.5	-78.3	0.1	7.0	1172.2	10788	-3.9	-8.9	-20.9	1.1	6.6	46.6
19	682	2.7	24.3	205.6	0.1	7.1	1129.4	4693	-0.2	-3.6	-17.6	1.3	7.5	52.8
20	1402	2.4	15.0	88.1	0.1	6.9	905.2	9110	4.4	10.4	25.9	1.2	6.8	47.0
21	675	0.3	-10.7	-324.7	0.1	6.8	1174.8	5335	1.6	1.9	0.3	1.2	7.4	54.6
22	701	1.4	8.5	48.5	0.1	6.8	1109.6	4500	2.5	6.1	15.0	1.2	6.7	46.9
all	52220	-0.3	1.2	74.1	0.1	7.2	1165.1	360839	0.5	1.8	5.7	1.2	6.6	46.4
