# Supplementary material for: Cohen’s h for detection of disease association with rare genetic variants
Source: BMC Genomics. 2014 Oct 8;15(1):875. doi: 10.1186/1471-2164-15-875 (PMC4198687; doi:10.1186/1471-2164-15-875)
Supplement: Supplementary file 3 — Additional file 3: Type I error rates for RD, Cohen’s h and log(OR) for 22 chromosomes. (PDF 91 KB) [file 12864_2014_6546_MOESM3_ESM.pdf]

Additional file 3: Type I error rates for RD, Cohen’s h and log(OR) for 22 chromosomes.

Chr	no. of rSNP	RD	Cohen's h	log(OR)	no. of cSNP	RD	Cohen's h	log(OR)
1	4399	0.048	0.052	0.045	28753	0.046	0.046	0.046
2	4341	0.053	0.057	0.049	30271	0.048	0.049	0.048
3	3484	0.050	0.052	0.048	25113	0.048	0.048	0.048
4	3506	0.049	0.054	0.046	23447	0.050	0.050	0.050
5	3462	0.056	0.059	0.052	24020	0.050	0.050	0.050
6	3138	0.064	0.068	0.061	24026	0.054	0.054	0.054
7	2641	0.048	0.050	0.044	19504	0.045	0.046	0.045
8	2835	0.042	0.049	0.042	20353	0.053	0.053	0.053
9	2514	0.050	0.054	0.046	17134	0.053	0.053	0.053
10	3205	0.052	0.056	0.050	20923	0.048	0.049	0.048
11	2656	0.056	0.066	0.053	19456	0.056	0.056	0.056
12	2722	0.059	0.063	0.054	18370	0.055	0.055	0.054
13	2124	0.065	0.067	0.060	14063	0.050	0.050	0.050
14	1759	0.043	0.047	0.039	11421	0.041	0.041	0.041
15	1610	0.052	0.058	0.050	10287	0.056	0.056	0.056
16	1563	0.038	0.042	0.036	11015	0.043	0.043	0.043
17	1160	0.051	0.055	0.045	8257	0.052	0.052	0.052
18	1641	0.046	0.052	0.043	10788	0.047	0.047	0.047
19	682	0.051	0.053	0.045	4693	0.053	0.053	0.053
20	1402	0.047	0.050	0.043	9110	0.050	0.050	0.050
21	675	0.046	0.049	0.044	5335	0.056	0.056	0.056
22	701	0.049	0.051	0.047	4500	0.045	0.045	0.045
all	52220	0.051	0.056	0.048	360839	0.050	0.050	0.050
