# Supplementary material for: Cohen’s h for detection of disease association with rare genetic variants
Source: BMC Genomics. 2014 Oct 8;15(1):875. doi: 10.1186/1471-2164-15-875 (PMC4198687; doi:10.1186/1471-2164-15-875)

Additional file 5: Box-plot of effect sizes for common SNPs based on two shared controls in WTCCC data. Panel A: RD; Panel B: Cohen's h; Panel C: log(OR).

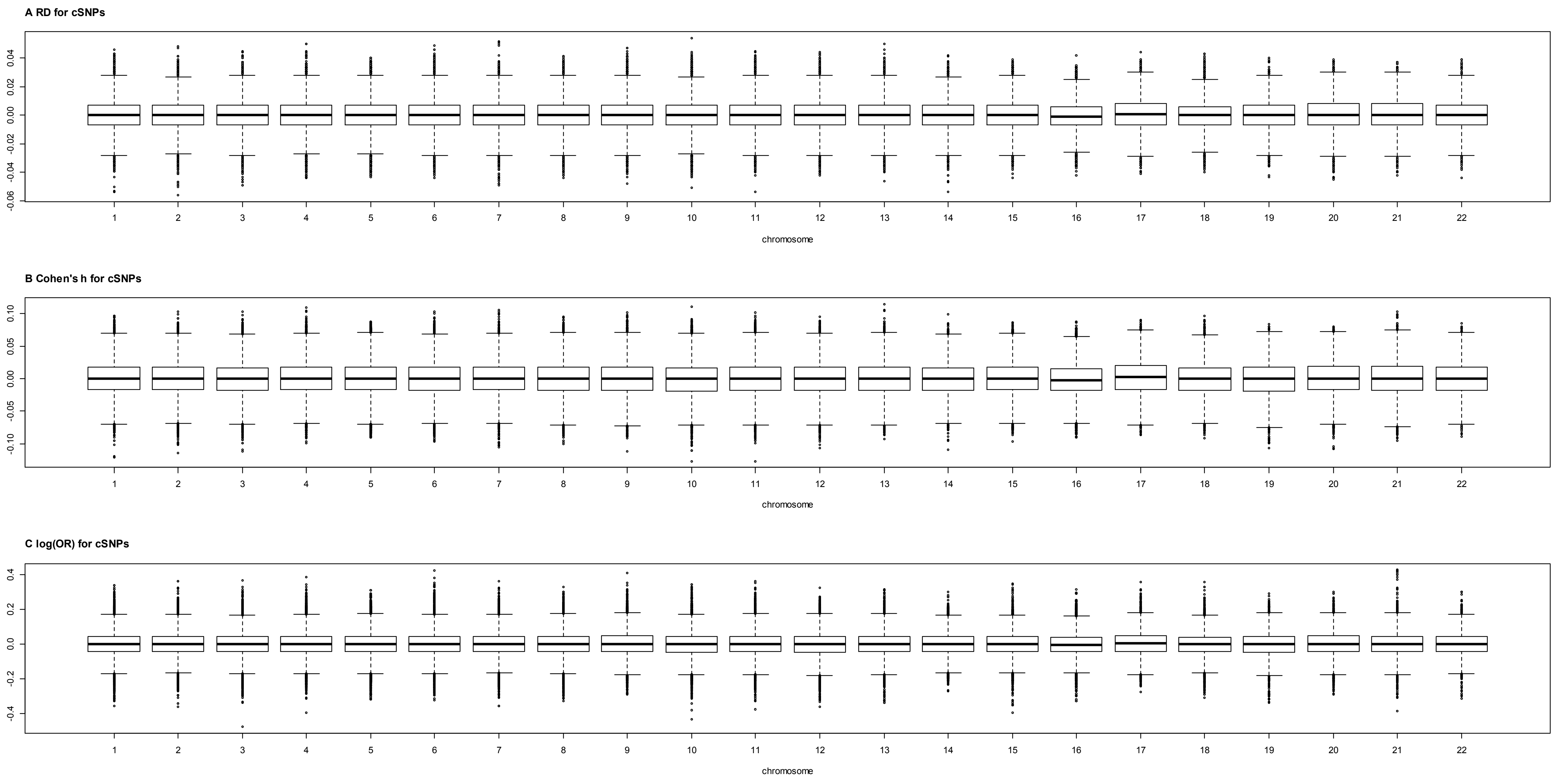

Supplement: Supplementary file 5 — Additional file 5: Box-plot of effect sizes for common SNPs based on two shared controls in WTCCC data. Panel A: RD; Panel B: Cohen’s h; Panel C: log(OR). (PDF 353 KB) [file 12864_2014_6546_MOESM5_ESM.pdf]
