# Supplementary material for: Cohen’s h for detection of disease association with rare genetic variants
Source: BMC Genomics. 2014 Oct 8;15(1):875. doi: 10.1186/1471-2164-15-875 (PMC4198687; doi:10.1186/1471-2164-15-875)

Additional file 6: Proportions of mild, moderate, and large effect for common SNPs in CAD. Grey bar represents OR and Black bar denotes Cohen's h.

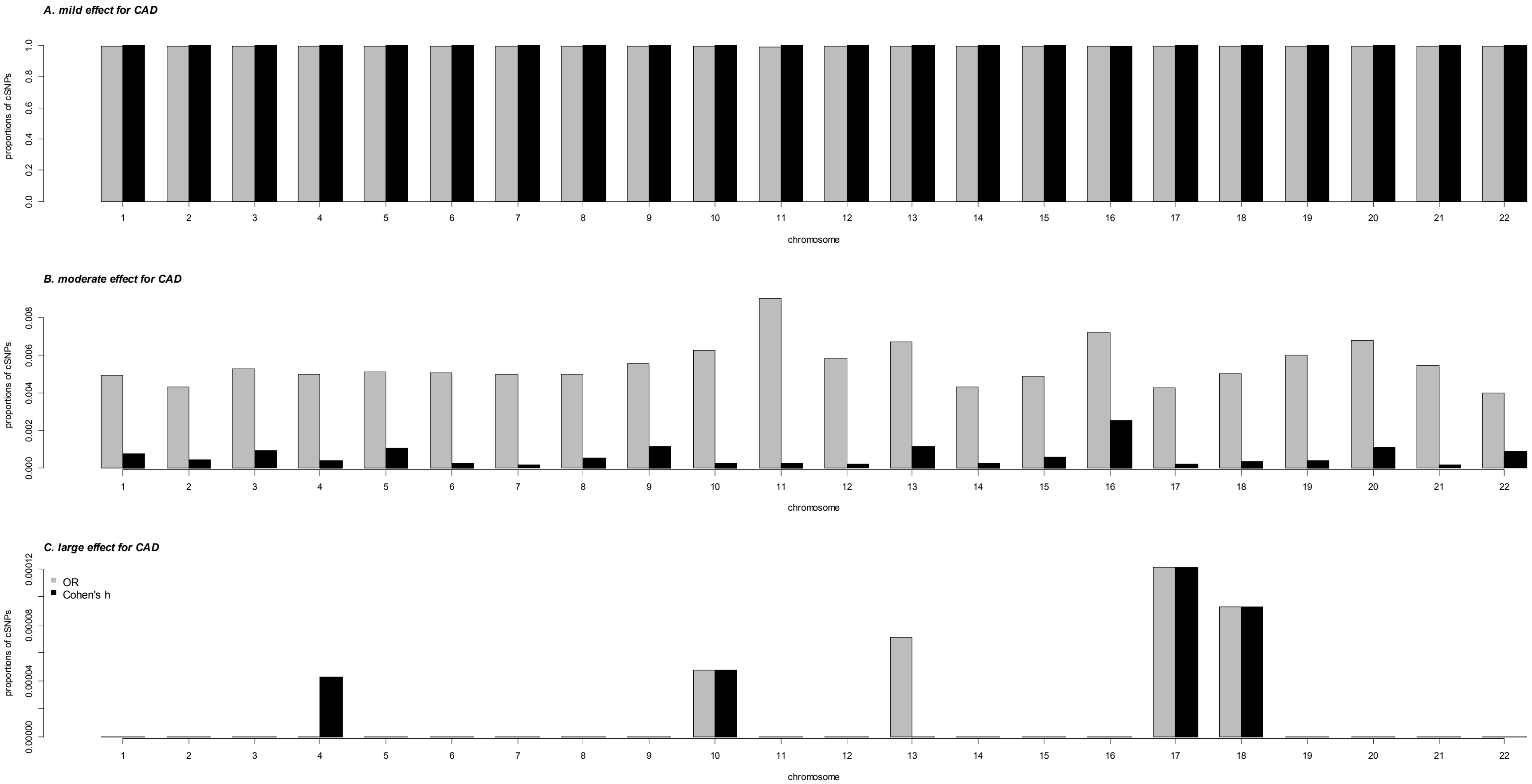

Supplement: Supplementary file 6 — Additional file 6: Proportions of mild, moderate, and large effect for common SNPs in CAD. Grey bar represents OR and Black bar denotes Cohen’s h. (PDF 254 KB) [file 12864_2014_6546_MOESM6_ESM.pdf]
